# Assessment of an Immuno-Diagnostic Method for Hookworm-Related Cutaneous Larva Migrans Using Crude Extracts of *Ancylostoma caninum*

**DOI:** 10.3390/tropicalmed8040209

**Published:** 2023-03-30

**Authors:** Sitthithana Adam, Paron Dekumyoy, Duangporn Nacapunchai, Thawatchai Ketboonlue, Prakaykaew Charunwatthana, Jittima Dhitavat, Khuanchai Koompapong, Putza Chonsawat, Dorn Watthanakulpanich

**Affiliations:** 1 Department of Helminthology, Faculty of Tropical Medicine, Mahidol University, 420/6 Ratchawithi Road, Ratchathewi, Bangkok 10400, Thailand; 2 College of Allied Health Sciences, Suan Sunandha Rajabhat University, Bangkok 10300, Thailand; 3 Department of Clinical Tropical Medicine, Faculty of Tropical Medicine, Mahidol University, 420/6 Ratchawithi Road, Ratchathewi, Bangkok 10400, Thailand; 4 Department of Protozoology, Faculty of Tropical Medicine, Mahidol University, 420/6 Ratchawithi Road, Ratchathewi, Bangkok 10400, Thailand; 5 Hospital for Tropical Diseases, Diagnostic Laboratory Unit, Faculty of Tropical Medicine, Mahidol University, 420/6 Ratchawithi Road, Ratchathewi, Bangkok 10400, Thailand

**Keywords:** *Ancylostoma caninum* antigen, total IgG, IgG subclasses, indirect ELISA, cutaneous larva migrans

## Abstract

People can become infected with cutaneous larva migrans (CLM) through skin penetration by the infective zoonotic larvae of hookworms. Few studies have investigated CLM’s immunodiagnosis, and the existing studies were limited to crude somatic or excretory/secretory antigens (Ags) from adult worms. Here, we aimed to develop an indirect enzyme-linked immunosorbent assay (ELISA) to differentiate and diagnose hwCLM by detecting immunoglobulin (Ig)E, IgG, and IgG subclasses 1–4 (IgG_1–4_) against the somatic Ag of adult *Ancylostoma caninum* checkerboard titrations of adult *A. caninum* worm extract. Pooled serum controls were immunocharacterized using an indirect ELISA. The IgG_1–4_ and IgE results were unsatisfactory; however, the use of total IgG achieved results comparable to those of immunoblotting. Thus, we continued to analyze the IgG-ELISA using serum samples from patients with hwCLM and heterologous infections as well as from healthy controls. The sensitivity and excellent specificity of the total IgG-ELISA were 93.75% and 98.37%, respectively, and its positive and negative predictive values were 75% and 99.67%, respectively. Antibodies from five cases of angiostrongyliasis, gnathostomiasis, and dirofilariasis cross-reacted with the somatic Ag of adult *A*. *caninum*. This new assay can adequately serodiagnose hwCLM when combined with clinical features and/or histological examination.

## 1. Introduction

Cutaneous larva migrans (CLM), also known as creeping eruption, sandworm eruption, plumber’s itch, and serpiginous dermatitis, is caused by the intradermal penetration and migration of several larvae of helminths, mainly canine hookworms (zoonotic hookworms) and also including *Ancylostoma caninum*, *A. braziliense*, and *A. ceylanicum*, which are well-known causative agents of hookworm-related CLM (hwCLM), and other helminths such as *Gnathostoma* spp., *Loa loa*, *Uncinaria stenocephala*, *Pelodera strongyloides, Strongyloides stenocephala*, *Dirofilaria repens*, *Fasciola* (ectopic feature), and *Schistosoma* may mimic similar migratory skin lesions [1,2,3,4,5,6]. HwCLM infections are mainly distributed across tropical and subtropical countries [2,4]. The species of zoonotic hookworms that infect humans vary throughout different regions [3,7]. Globally, 1.3 billion people are infected by hookworms, and about 878 million school-age children are at risk, according to the WHO. In addition, hookworm infection can lead to approximately 65,000 deaths annually, thereby resulting in 845 thousand DALYs (disability-adjusted life years) per year. The hw infection here focuses on *N. americanus* and *A. duodenale*; however, *A. caninum* zoonotic hookworms also develop into adults in humans [8]. Definitive hosts, dogs and cats, are the main CLM transmitters to humans, and several hookworm infection studies reported their prevalence in different regions, such as 77 of 80 dogs infected with *A. caninum* (96.3%) and *A. braziliense* (49.4%) in Uruguay [9], 19% of 63 stray dogs infected with *A. braziliense* and 27% infected with *A. caninum* in South Africa [10], and 66.3% of 178 dogs infected with *A. caninum* in China [11]. In humans, a report of infected travelers stated that 98 patients with hwCLM visited Southeast Asia (31 patients, 31.6%), the Caribbean or Central America (27, 27.6%), South America (13, 13.4%), East Africa (10, 10.2%), the Indian Subcontinent (10, 10.2%), West Africa (5, 5.1%), South Africa (1, 1%) and North Africa (1, 1%) [12]. The larvae of zoonotic hookworms infect humans by penetrating the skin via contaminated soil, sand on the ground, or consumption of larvae on grass/vegetables. HwCLM demonstrates its clinical symptoms on the skin of humans in the epidermis and infrequently in the upper dermis [7,13,14,15,16,17,18]. Symptoms develop within a few days after larval penetration but often requires only symptomatic treatment, even for severe cases. Moreover, other organs and tissues are also reported to be affected by rare infections, such as those in the lungs, including migratory pulmonary infiltrates and Loeffler’s syndrome, in which the larva in the sputum was found to possibly be *A. braziliense* or *A. caninum* [19,20]; the clinical symptoms involved are coughing with green sputum and erythematous eruption on the palate after presenting complicated symptoms and after having serpiginous eruption on the feet [21]. Mouth infections present in the tongue, lips, cheeks, floor of mouth, palate, and oral mucosa oropharynx [22,23,24,25,26]. Reported small intestinal infections include those by *A. braziliense* larvae [27], visceral larval migrans manifesting as hepatomegalia caused by *A. caninum* in a child [28], and those probably caused by *A. caninum* larvae in skeletal muscle fiber [29] and in corneas [30,31]. In addition, the sporadic infections by adult *A. caninum* in human intestine were reported in the Philippines, South America, and Israel [32,33,34,35,36].

Methods for diagnosing hwCLM are usually based on the clinical presentation of pruritus and erythematous scaly lesions as well as a history of recent travel to tropical or subtropical regions with exposure to a beach or jungle [12,37]. However, misdiagnosis and inappropriate treatment (58%) were found in patients vacationing in tropics or subtropics [38] in which zoonotic hookworms could possibly infect other tissues and organs of the infected patients, not the skin, as mentioned previously. Few publications have reported on the developing serodiagnostic tests for human zoonotic hookworm infections [12,13,38,39,40,41]. An IgG-ELISA based on the *A. caninum* excretory-secretory antigen was attempted in eosinophilic enteritis cases, and its efficacy was proven [40,42]. Human hookworm (*A. duodenale*)-infected patients were diagnosed by ELISA using an animal hookworm (*A. caninum*) larvae antigen, which gave higher results in IgG- and IgG_4_-ELISAs than when using a *N. americanus* adult antigen [43]. Clinical presentation and patient history alone may not be enough to predict these infections because inexperienced physicians misdiagnose these parasites as those that cause similar symptoms, including larvae and animal hookworm itself in ectopic foci. Therefore, we aimed to develop an indirect enzyme-linked immunosorbent assay (ELISA) to differentiate and diagnose hwCLM from other CLM etiologies by detecting IgE, IgG, and IgG subclasses 1–4 (IgG_1–4_) against the somatic Ag of adult *A*. *caninum*.

## 2. Materials and Methods

### 2.1. Antigens

Twenty female and twenty male adult *A. caninum* worms were morphologically identified and stored at −80 °C for seventeen years at the Immunodiagnostic Unit for Helminthic Infections, Department of Helminthology, Faculty of Tropical Medicine, Mahidol University (Bangkok, Thailand). Prior to analysis, the worms were washed with normal saline solution and distilled water and homogenized using a mortar and pestle with distilled water and alumina in an ice tray. The homogenate was sonicated with an ultrasonicator (Ultrasonic Processor XL; Qsonica, LLC, Newtown, CT, USA) at 1-min intervals for 10 min and then centrifuged (Eppendorf AG, Hamburg, Germany) at 18,000× *g* at 4 °C. Afterward, the supernatant was collected to determine its protein content using the Pierce™ Bicinchoninic Acid (BCA) Protein Assay Kit (Thermo Fisher Scientific, Waltham, MA, USA). Then, the supernatant was stored at −80 °C until used. The quality of the crude somatic extract was analyzed by using gel electrophoresis and staining.

### 2.2. Serum Samples

**Figure 1:**
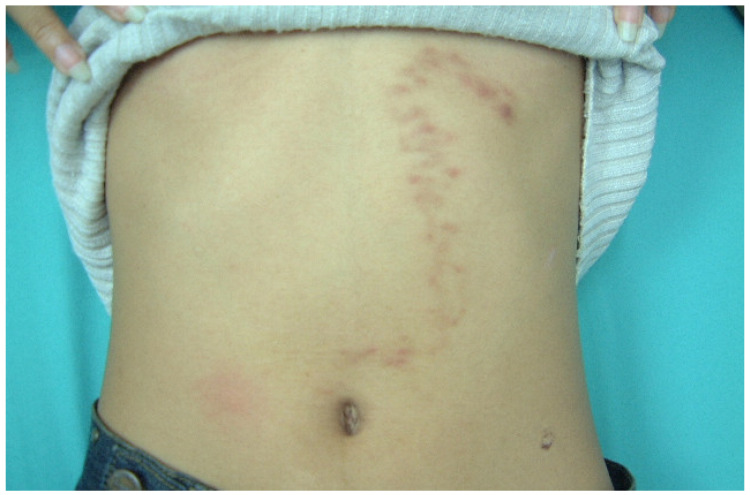
Hookworm-related cutaneous larva migrans of a female patient presenting a zigzag track on the abdomen.

**Figure 2:**
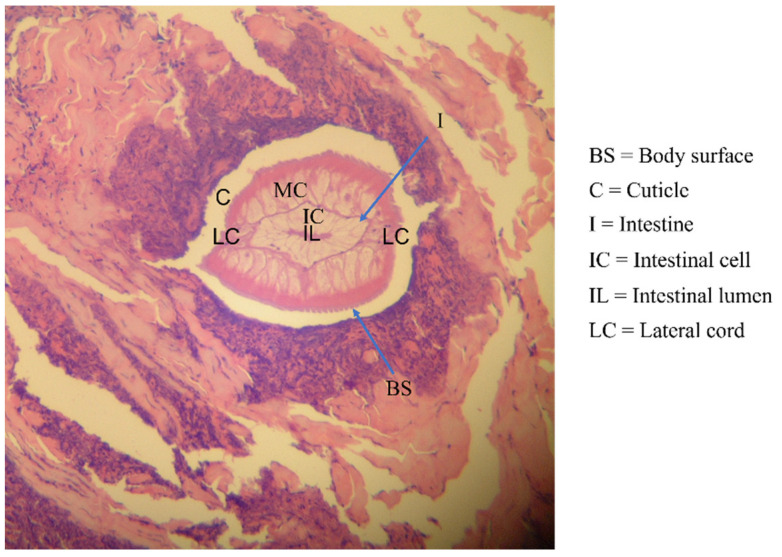
Section of biopsied larva from the abdomen showing the cuticle, body surface, intestine-containing cavity, several intestinal cells, and muscle cell lateral cords.

**Figure 3:**
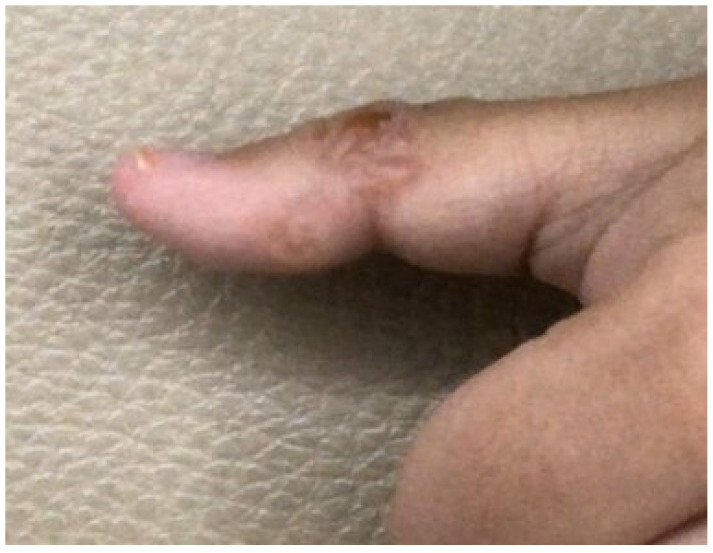
Hookworm-related cutaneous larva migrans on the thumb of the right hand of a young male patient showing a thread-like line of serpigenous inflammation.

The ethical committee of the Faculty of Tropical Medicine, Mahidol University, approved the use of human sera in this study (Ethical Approval No. MUTM 2014-071-01). Serum samples (*n* = 326) were stored as antibody stocks at −80 °C for between 3 and 34 years at the Immunodiagnostic Unit, Department of Helminthology, Mahidol University. The serum samples were divided into the homologous hwCLM sera (*n* = 16), heterologous sera with various helminth and protozoan infections (*n* = 280), and healthy sera (*n* = 30) groups. Table 1 and Figure 1, Figure 2 and Figure 3 provide additional information on some hwCLM samples.

### 2.3. ELISA

**Figure 4:**
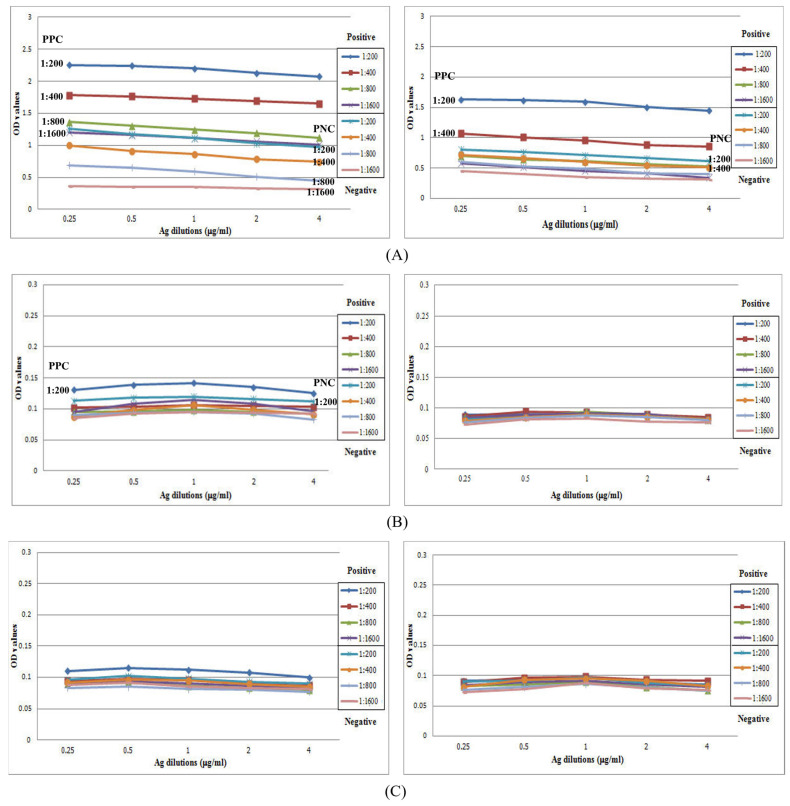

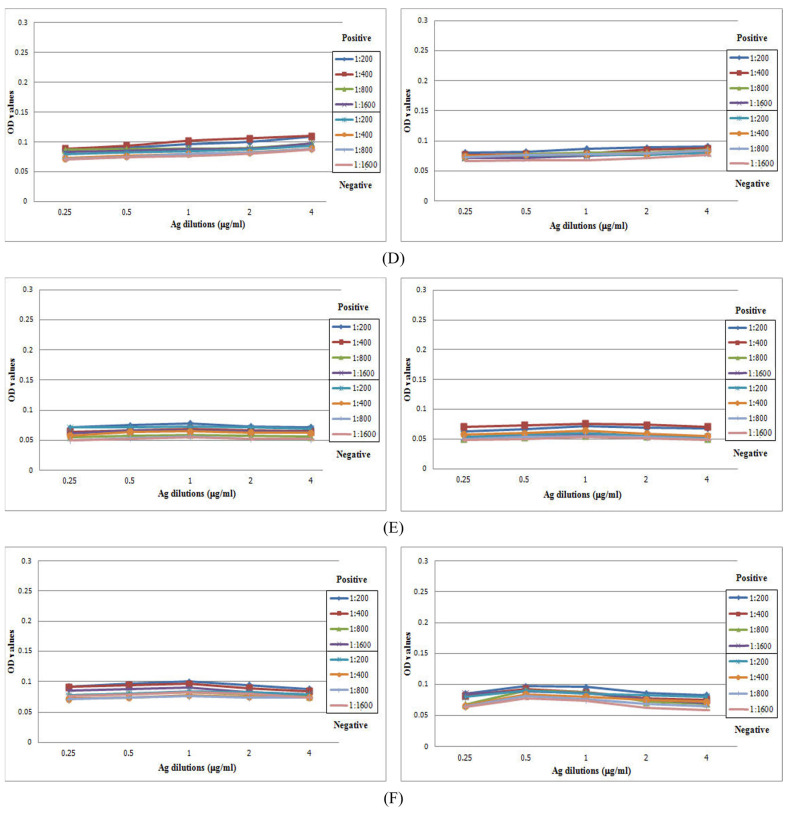
Checkerboard titration curves for determining the optimal concentration of *A. caninum* crude antigens (0.25–4 μg/mL) against various dilutions of pooled positive controls (PPC) and pooled negative controls (PNC) using 1:2000 (L, Left) and 1:4000 (R, Right) dilutions of peroxidase-conjugated anti-human IgGs ((**A**): IgG, (**B**): IgG_1_, (**C**): IgG_2_, (**D**): IgG_3_, (**E**): IgG_4_, and (**F**): IgE). Lines represent OD values of dilutions of either pooled positive serum or pooled negative serum at ratios of 1:200, 1:400, 1:800, and 1:1600.

A checkerboard titration method with a twofold condition was used to optimize the indirect ELISA protocol. Immunocharacterizations of the ELISA checkerboard titration curves of the optical density (OD) values showed that total IgG had the best separation between positive and negative pooled controls, but the IgG_1–4_ and IgE curves were unsatisfactory (Figure 4). Therefore, only total IgG was used for the ELISA, carried out in accordance with the procedure of Yoonuan et al. [46]. The optimal ELISA conditions were 0.25 µg/mL *A. caninum* Ag in 0.05 M carbonate–bicarbonate buffer at pH 9.6 with a serum dilution of 1:1600 and an anti-human IgG conjugate dilution of 1:2000. Briefly, the wells of a 96-well plate were coated with the Ag, washed, and blocked with 1% skim milk- and 0.02% NaN_3_ in phosphate-buffered saline (PBS). Each diluted serum sample reacted with Ag at 37 °C for 1.5 h. After washing, the immunoreaction was combined with diluted anti-human IgG Ab conjugated with horseradish peroxidase (SouthernBiotech, Birmingham, AL, USA). After incubating the plate at 37 °C for 1.5 h, the reaction was visualized via incubation for 30 min with ABTS substrate solution (2,2-azino-di-[3-ethyl-benzthiazoline sulfonate]; Sigma-Aldrich Canada Co., Oakville, ON, Canada). The reaction was stopped by adding 1% sodium dodecyl sulfate (SDS), and absorbance was measured using a microplate reader at a wavelength of 405 nm.

### 2.4. Observation of Crude Extract Profiles via Staining and Antibodies

Because the worms were stored at −80 °C for several years, ice crystals may have damaged the internal structures of the frozen worms and proteins during processing, which involved various temperatures from −12 °C to −75 °C [47,48]. Somatic extract patterns were demonstrated by transferring 15 and 30 µg of the extract into SDS-polyacrylamide gel via electrophoresis (ATTO Corporation, Tokyo, Japan) with 5% stacking and 13% separating gels, which was followed by simple staining. The two extract concentrations were treated with sample buffer (1.5×; 0.25 M Tris-HCl, pH 6.8, 1.75% SDS, 3.75% mercaptoethanol, 7.5% glycerol, and 0.008% bromophenol blue) at 100 °C for 3 min in a heat block (Accublock™; Labnet International Inc., Edison, NJ, USA); then, the proteins were separated via SDS-PAGE at a constant current of 20 mA, performed in accordance with the protocol of Dekumyoy et al. [49] with slight modifications. Finally, the gel was stained with Coomassie brilliant blue and destained.

As the IgG_1–4_ and IgE checkerboard titration results were unsatisfactory (Figure 4), we performed immunoblotting [49] to observe the reactions between the Ag and IgG_1–4_ and IgE. A blot of the somatic extracts was analyzed based on the Ab reactions. The electrophoresed extracts (30 µg) were transferred to a nitrocellulose membrane using a semi-dry transfer cell (ATTO Corporation). The nonbinding sites on the membrane were blocked with 3% skim milk- 0.02% NaN_3_ in PBS for 1 h on a rocking platform. Each strip was incubated in a 1:50 dilution of pooled positive serum from the hwCLM samples in 0.02% NaN_3_-PBS-Tween20 (PBS-T) overnight on a rocking platform. The strips were then incubated with individual anti-human Abs against total IgG, IgG_1–4_, and IgE (SouthernBiotech) and conjugated with horseradish peroxidase at a dilution of 1:1000 in PBS-T for 2 h. Afterward, the membrane strips were treated with the substrate solution (2,6-dichlorophenol indophenols)-H_2_O_2_. The reaction was stopped by washing them with distilled water, and the Ag–Ab complex formations were observed.

### 2.5. Data Analysis

The cut-off values of the indirect ELISA were determined via analysis of a receiver operating characteristic curve (95% confidence interval) using PASW Statistics for Windows, version 18.0. (SPSS Inc., Chicago, IL, USA). Sensitivity, specificity, and predictive values were calculated using the 2 × 2 table method [50]. Scatter plots of the OD values obtained from the indirect ELISA were created using GraphPad Prism, version 6.00 for Windows (GraphPad Software, Inc., La Jolla, CA, USA).

## 3. Results

### 3.1. Analysis of Crude Extract Profiles

**Figure 5:**
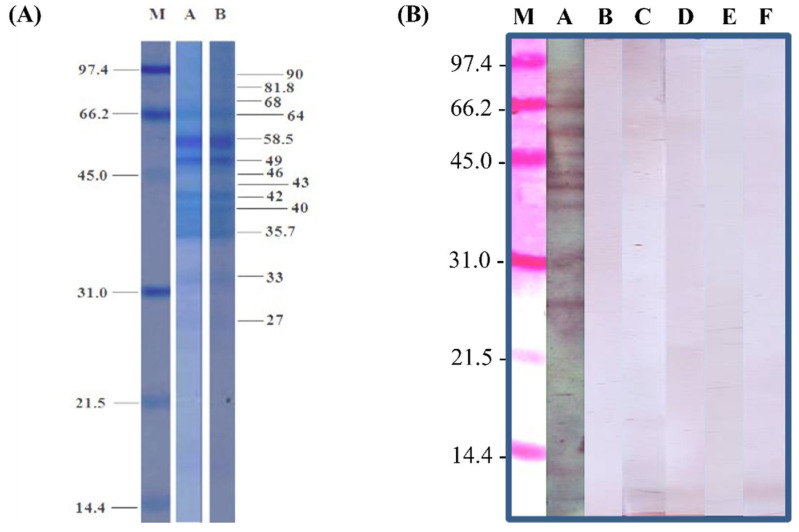
(**A**) Protein profile comparison of *A. caninum* crude extracts at 15 (A) and 30 (B) µg/well shown by Coomassie brilliant blue staining. M: low molecular weight markers (kDa). Demonstration of strong bands at 58.5 and 49 kDa and faint bands from 90 to 27 kDa. (**B**) Anti-human immunoglobulins IgG, IgG_1–4_, and IgE reacted with pooled human antibodies of hwCLM against crude *A. caninum* somatic antigens via immunoblot. M: low molecular weight markers; A: IgG; B–E: IgG_1–4_; F: IgE. Reaction of total IgG (lane A) was strong in a range of standard markers from 97.4 to 21.5 kDa and below 14.4 kDa. IgG_1–4_ (lanes B–E) and IgE (lane F) showed weak and undefined bands, with more bands in IgG_2_ (lane C) and IgG_3_ (lane D) in a range of markers > 45 kDa and weak bands below 14.4 kDa in lanes C, D, and E (IgE). Several weak and smeared bands were found in all reactions of IgE and IgG_1–4_.

The concentration of the extract containing both female and male worms was 1 mg/mL. For SDS-PAGE protein analysis, 15 and 30 µg of the extract demonstrated good quality with defined protein bands after Coomassie brilliant blue staining. Most of the protein profiles had many distinct bands with molecular weights of >31 kDa and relatively few bands with molecular weights of <31 kDa. Strong bands were detected at 64, 58.5, 49, 42, 40, 35.7, and 33 kDa (Figure 5A).

### 3.2. Correlations between Immunoglobulins and Antigens

After the checkerboard titrations (Figure 4), the OD value curves of the total IgG from the pooled positive controls (hwCLM) differed from those of the pooled negative controls in the same dilutions. Twofold antigen concentrations of 0.25−4 μg/mL, a 1:2000 dilution of the secondary Ab, and a 1:200 dilution of hwCLM serum showed high OD values ranging from 2.0 to 2.5. The 1:1600 dilution showed OD values ranging from 1.0 to 1.5 on a Y scale. The negative control diluted at 1:200 showed high OD values ranging from 1.0 to 1.5. The negative control diluted at 1:1600 showed OD values ranging from 0 to 0.5 on a Y scale (Figure 4A). For IgG_1–4,_ low OD values of each dilution of the pooled positive (hwCLM) and negative controls were similar and ranged from 0.05 to 0.15. The OD values of all diluted sera from both the pooled positive (hwCLM) and negative controls did not separate from each other at those antigen concentrations (Figure 4B−E). For IgG_1_ and IgG_2_ at the OD values of the 1:200 dilutions, the pooled positive control (hwCLM) values were less separated from the negative control values following twofold antigen dilution. For IgE, its OD value was separated in the same manner as the IgG_1–4_ values between the pooled positive and negative controls and ranged from 0.05 to 0.10. After separating the OD values, IgG was further analyzed at an Ag concentration of 0.25 μg/mL, a serum dilution of 1:1600, and a secondary Ab dilution of 1:2000. The curves of the Ag–Ab complexes from checkerboard titration could not be appropriately differentiated from the pooled positive and negative serum controls when analyzed using IgG_1–4_ and IgE. The reaction curves were in groups for all serum dilutions and antigen concentrations. Additionally, the Ag–Ab complexes could not be appropriately differentiated via immunoblot analysis. The reactions produced many strong distinct reactive bands for total IgG but not for IgG_1–4_ or IgE (Figure 5B). This thereby demonstrates the poor IgE and IgG_1–4_ reactions of the positive pooled serum controls at 1:200 to 1:1600 dilutions in the checkerboard titrations via immunoblot.

### 3.3. Full Scale IgG-ELISA

**Figure 6:**
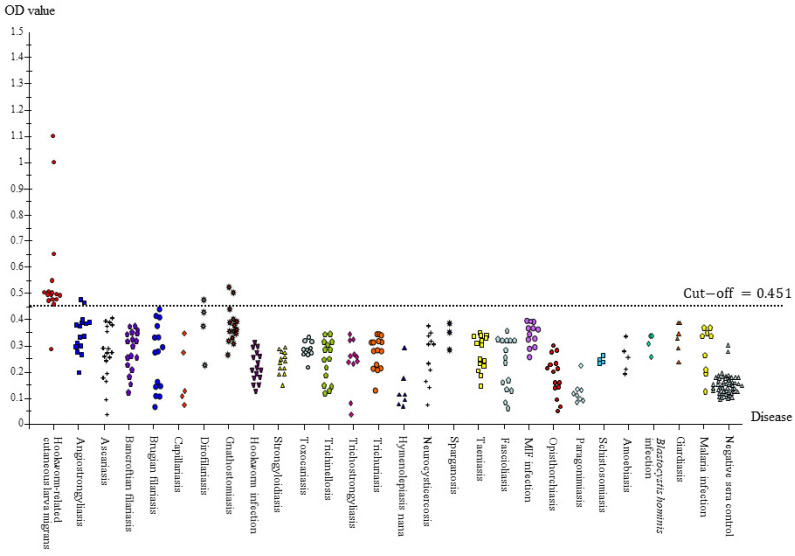
Scatter patterns of IgG-ELISA absorbance values of serum samples from hookworm-related CLM, 27 different diseases, and negative control against *A. caninum* crude antigen. Ag–Ab reactions were accomplished under ELISA conditions, 0.25 μg/mL of Ag concentration, 1:1600 diluted serum samples, and an anti-human IgG dilution of 1:2000 at a cut-off value of 0.451.

All serum samples were subjected to IgG-ELISA. Of the 16 hwCLM samples, 1 was a false negative, and the OD values of the other 15 samples ranged from 1.111 to 0.452 (cut-off value: 0.451). Most of the hwCLM OD values ranged from 0.452 to 0.556. The false negative case had an OD value of 0.286. There were two high OD values in the hwCLM cases, 1.111 and 1.078, which separated them from the group. Therefore, the sensitivity and specificity of the IgG-ELISA were 93.75% and 98.39%, respectively, and its positive and negative predictive values were 75% and 99.67%, respectively. Only three nematodiases yielded false positives toward the crude adult *A. caninum* Ag, i.e., angiostrongyliasis (2/15), gnathostomiasis (2/15), and dirofilariasis (1/4). Nonetheless, we achieved satisfactory results because we found no cross-reactions in the serum samples for 24 diseases: nematodiases (ascariasis, capillariasis, Bancroftian filariasis, Brugian filariasis, hookworm infection, strongyloidiasis, trichostrongyliasis, toxocariasis, trichinellosis, and trichuriasis), cestodiases (taeniasis saginata, neurocysticercosis, cystic echinococcosis, sparganosis, and hymenolepiasis nana), trematodiases (paragonimiasis, opisthorchiasis, fascioliasis, minute intestinal fluke infection, and schistosomiasis), and protozoan infections (amoebiasis, giardiasis, *Blastocystis* infection, and malaria) (Figure 6). It was found that strongyloidiasis (11 cases), fascioliasis (3 cases), and schistsomiasis (4 cases) can cause CLM symptoms, but no false positive occurred. Negative control sera demonstrated good discrimination from the hwCLM cases; all negative control sera yielded true negative results, and only two samples were less distinct from others in this group.

## 4. Discussion

When focusing on IgG, several previous studies assessed total IgG but yielded poor results. In contrast, the selected crude *A. caninum* somatic Ags after our checkerboard titration reacted less with Abs against cestodiasis, trematodiasis, and protozoan infections, including ten nematode-associated diseases, and all OD values were below the cut-off value. This may be because of the small amount of the Ags used (0.025 μg/100 μL of coating buffer) and their low antigenicity to the Abs in those tested serum samples. Finally, our proposed ELISA conditions for evaluating IgG yielded 93.75% sensitivity and 98.39% specificity. All healthy control sera had low OD-ELISA values in response to the Ags and yielded similar OD values.

One hwCLM sample in our study had few IgG Abs to *A. caninum* somatic Ag; thus, it gave a false negative result. This patient had a clinical history of subcutaneous swelling and other erythematous skin changes and larval migrans, and his sero-tests were negative for gnathostomiasis and strongyloidiasis. The gnathostomiasis test was done via detection of a 24-kDa diagnostic band of the *G. spinigerum* Ag on an IgG immunoblot, and the strongyloidiasis test was performed via IgG-ELISA using a molecular weight cut-off Ag of *Strongyloides stercoralis* infective larvae; the test was performed at the Department of Helminthology, Faculty of Tropical Medicine. In 2003, a previous study of a patient showed clinical features of erythematous linear and serpiginous plaques. The patient tested positive for crude *A. caninum* Ags and negative for *G. doloresi* Ags using the IgG-ELISA. Researchers attempted to screen this patient’s antibodies for other infections using a multiple-dot ELISA with 12 Ags: *Dirofilaria immitis*, *Toxocara canis*, *Ascaris suum*, *Anisakis simplex*, *G. doloresi*, *Strongyloides ratti*, *Paragonimus westermani*, *P. miyazakii*, *Fasciola hepatica*, *Clonorchis sinensis*, *Spirometra erinacei*, and *Cysticercus cellulosae*. The tests were negative, and the patient was diagnosed with *A. caninum* infection [7]. Additionally, the sensitivity and specificity of the proposed ELISA for diagnosing a single case of hwCLM in that study were suboptimal.

In our study, *A. caninum* somatic Ag was screened and did not cross-react with Abs from 24 parasitic diseases or with the negative controls. Only five sera samples from three nematodiasis patients cross-reacted with *A. caninum* somatic Ag. These false positives may not be encountered for serodifferentiation if the targeted habitats of those helminths are considered, i.e., *Angiostrongylus* worms mostly infect the brain. *Dirofilaria immitis* exists as nodules in the lungs or tissues because humans are the dead-end host for this worm, and *Gnathostoma* worms travel throughout the human body, organs, and especially the cutaneous tissues. All hwCLM cases in the present study were negative for gnathostomiasis (Table 1).

The larval and immature stages of many helminths, including *Gnathostoma*, *Strongyloides*, *sparganum*, *Paragonimus*, and *Fasciola*, can cause CLM cases similar to those of hookworms [2,5,51,52,53,54,55], but Abs against these helminths in our study did not cross-react with the *A. caninum* somatic Ag. Additionally, a few Abs in two of fifteen cases of gnathostomiasis showed false positives. However, animal hookworms mostly migrate in the dermis of human skin, but *Gnathostoma* worms can move in the skin and into deeper tissues and organs.

After several years of frozen storage, the worms were not degraded and demonstrated sharp and defined protein bands on Coomassie brilliant blue-stained gel. Ice crystal damage did not appear to affect the frozen worms. However, the adult worm Ag in our study did not react well with the pooled positive and negative control sera on the ELISA checkerboard titration for individual IgG subclasses and IgE, which is possibly because the serum samples from the controls contained low levels of individual immunoglobulins. The duration of storage of serum samples at −80 °C did not affect antibody reactivity because two high OD values, 1.111 and 1.078, were from two young boys whose sera were kept for 34 and 3 years, respectively. The false negative case was an adult patient, and his serum sample was kept for 11 years. To support low IgE in hwCLM, IgE is produced after all other isotypes in the blood [56], with the lowest concentration among serum Igs of a sequential Ig response [57]. Some reports of hookworm-related CLM presented laboratory findings with normal serum IgE levels [14,39], and the Ags from canine hookworm larvae were mostly confined to the dermis of humans (an abnormal host), thereby revealing a limited IgE response to infection [12]. Hence, the low or absent IgE levels in the positive controls for hwCLM in our study may have led to their being indistinguishable from the negative controls in the checkerboard titration. The increasing IgE levels may have depended on the magnitude of infection such as in schistosomiasis, filarial tropical eosinophilia [58], and *N. americanus* self-infection, which requires three to four infections to measure increases in IgE [59]. Helminth species other than *A. caninum* can cause increased IgE levels because their locations are not limited to the dermis [12].

Finding a different result when comparing antigens from *A. caninum* worms, Loukas and colleagues [13] used a different Ag from *A. caninum* worms, excretory-secretory (ES) Ag, against four helminthiases, which did not react with Abs from human hookworm infections but reacted with Abs to strongyloidiasis, toxocariasis, and schistosomiasis. In our study, crude *A. caninum* somatic Ag did not cross-react with Abs against human-associated hookworm diseases, including strongyloidiasis, toxocariasis, and schistosomiasis, which differs from the study using ES-Ag. In Thailand, *N. americanus* is mainly reported via molecular confirmation, and minor species include *A. duodenale* and *A. ceylanicum* in humans at the studied sites [60,61]. Human-associated hookworms (15 sera used) in our study were confirmed only via detection of eggs and larvae; however, Abs from those hookworm infections may be induced by the aforementioned three species. Antibodies of those fifteen cases did not cross-react with the *A. caninum* Ag and thereby yielded true negative results (Figure 6). When comparing the crude somatic and crude ES Ags of *A. caninum*, Loukas and colleagues used an IgG-ELISA to study Abs from human eosinophilic enteritis with *A. caninum* infections and found a higher detection rate using ES-Ag than using a crude somatic Ag, although cross-reactions still occurred with parasitological soils [40]. Improvement of the diagnostic method using immunoblot with Ac68 of the ES product from *A. caninum* Ag detected eight ancylostomiasis caninum cases, five of which were positive for IgG and IgG_1–4_, one was positive for IgG and negative for IgG_1–4_, one was negative for IgG and positive for IgG_1–4_, and one was negative for all IgGs. All IgGs showed 75% sensitivity in immunoblot results, and IgG_4_ showed the best specificity (100%), which was determined from only three helminthiases (strongyloidiasis, trichinellosis, and schistosomiasis), but all human-associated hookworm infections (*A. duodenale*, *N. americanus* and unknown species) reacted with the Ac68 Ag. These authors found that Ac68 Ag was hookworm-specific [41].

Occasionally, when a serodiagnosis from a total IgG-ELISA cannot be interpreted because of high cross-reactivity, IgG_1–4_ can be analyzed to obtain a better diagnosis. However, previous studies recommended using an individual IgG subclass or co-interpretation of IgG subclasses for serodiagnosis, such as for gnathostomiasis (i.e., co-interpretation of IgG_1_-screening tests and IgG_2_-confirmed diagnosis via ELISA using the CSAg of *Gnathostoma* larvae) [62]. Laummaunwai and colleagues [63] reported that the highest sensitivity for immunoblotting for diagnosing gnathostomiasis using crude *Gnathostoma* larvae Ag was obtained with total IgG rather than IgG_1–4_, although specificity with IgG_4_ was slightly better than with total IgG (93.9% vs. 91.6%, respectively). HwCLM may be difficult to diagnose because of misdiagnoses and prescribed treatments from physicians and laboratory practitioners [64,65]. A previous study reported a misdiagnosis and improper treatment rate of 58% [38], including the observation of reported information over a long duration of increasing Igs because patients often visit a physician at the early onset of clinical symptoms. Thus, our results suggest that IgG_1–4_ and IgE from hwCLM sera cannot be used as mediators between diagnoses using ELISA and immunoblotting. However, the crude somatic Ag used in the present study had good sensitivity and excellent specificity once the optimal conditions for the IgG-ELISA were determined. Use of a crude Ag can demonstrate cross-reactivity with Abs of heterologous diseases; thus, proper ELISA conditions can improve the results.

## 5. Conclusions

Serodifferentiation with crude *A. caninum* somatic Ag for the proposed IgG-ELISA can potentially be used to diagnose hwCLM from *A. caninum* hookworms in humans. However, IgE and IgG_1–4_ could not be differentiated via the ELISA for the positive and negative controls following checkerboard titration; also in addition, the antigen–antibody banding pattern via immunoblot used pooled positive controls. Further studies are warranted to evaluate the proposed ELISA using more hwCLM cases and incorporating a purification technique during Ag preparation.

## Figures and Tables

**Table 1:** Descriptions of serum samples of 27 diseases and from healthy controls using different diagnostic methods, including clinical pictures of hwCLM cases.

Disease	Number of Serum Samples	Specific or Proven Diagnosis
**Homologous Sera**		
Human-related cutaneous larva migrans (hwCLM)	16	Clinical manifestations related to cutaneous larva migrans or creeping eruption, such as pruritus, swelling pain, and the appearance of an erythematous track on the skin of the trunk, buttock, hand, leg, etc. History of traveling or/and exposure to soil or beach land where dogs were around Tested negative for gnathostomiasis with an IgG-immunoblotting test and for strongyloidiasis with an IgG-ELISA, including a demonstration of two hwCLM cases and a section of larva structure from the patient via tissue biopsy (Figure 1, Figure 2 and Figure 3)
**Heterologous sera Nematodiasis**		
Angiostrongyliasis	15	Ocular angiostrongyliasis, larval stage found Larva in CSF sample found Consumption of snail intermediate hosts, stiff neck, and positive immunoblot
Ascariasis	18	Eggs in feces found by using the Kato thick smear technique
Bancroftian filariasis	17	Microfilariae on blood films
Brugian filariasis	15	Microfilariae on blood films and positive ELISA results
Capillariasis	5	Eggs and adults in feces found by using the simple smear technique and simple sedimentation
*Dirofilaria* infection	4	Sectioned worms in lung tissues and worms from the eyes and/or positive immunoblot results
Gnathostomiasis	15	Detection of worms and positive immunoblot results
Hookworm infection	15	Eggs in feces found by using the Kato thick smear technique or detection of L3 by using the polyethylene tube culture method
Strongyloidiasis	11	Larvae found in polyethylene tube culture
Toxocariasis	10	Clinical symptoms and positive immunoblot results
Trichinellosis	15	Biopsy and/or history of eating meat of wild pigs, clinical symptom manifestation, and positive immunoblot results
Trichostrongyliasis	11	Culture technique and larvae confirmation
Trichuriasis	15	Eggs found by using the Kato thick smear technique
**Cestodiasis**		
Cystic echinocossosis	6	Scolices found or positive immunoblot results; 1 indigenous case and 5 imported cases
Hymenolepiasis nana	8	Eggs in feces found by using the simple smear technique
Neurocysticercosis	11	Biopsy or clinical manifestation accompanying a computerized axial tomographic (CAT) scan of the brain and positive immunoblot results
Sparganosis	3	Spargana found
Taeniasis	15	*Taenia saginata* segments or *Taenia* eggs found in feces
**Trematodiasis**		
Fascioliasis	3	Eggs found or positive immunoblot results; 1 indigenous case and 2 cases supported by US Naval Medical Research Unit No. 3, Egypt
Opisthorchiasis	15	Worms detected post-treatment
Paragonimiasis heterotremus	15	Eggs in feces and sputum found by using the simple smear technique
Schistosomiasis	4	*Schistosoma mansoni* eggs found; cases supported by US Naval Medical Research Unit No.3, Egypt
Small intestinal fluke infection	10	Worms detected post-treatment
**Protozoa infections**		
Amoebiasis	5	Cysts in feces found by using the simple smear technique
*Blastocystis hominis* infection	4	Cysts in feces found by using the simple smear technique
Giardiasis	6	*Giardia intestinalis* cysts in feces found by using the simple smear technique
Malaria	9	*Plasmodium falciparum* or *P. vivax* found in blood smears
**Negative control sera**	30	Negative results from stool examinations using the simple smear and formalin-ether concentration techniques for other parasitic infections. Negative screening results from IgG-ELISA using ten antigens, namely crude antigens of *Gnathostoma spinigerum* larvae, *Strongyloides stercolaris* larvae, *Toxocara canis* male and female adult worms, *Angiostrongylus cantonensis* adult worms, *Dirofilaria immitis* female adult worms, *Trichinella spiralis* muscle larvae, *Taenia solium* metacestode, *Paragonimus heterotremus* worms, *Fasciola gigantica* worms, and fluid of hydatid cyst [44,45]

## Data Availability

Not applicable.
